# Modern Total Knee Arthroplasty Bearing Designs and the Role of the Posterior Cruciate Ligament

**DOI:** 10.1016/j.artd.2023.101130

**Published:** 2023-04-21

**Authors:** Kamran Movassaghi, Arpan Patel, Zohal Ghulam-Jelani, Brett R. Levine

**Affiliations:** a Department of Orthopaedic Surgery, University of California, San Francisco Fresno, Fresno, CA, USA; b Department of Orthopaedic Surgery, Rush University Medical Center, Chicago, IL, USA

## Abstract

The role of the posterior cruciate ligament (PCL) in total knee arthroplasty (TKA) surgery continues to be a source of debate among the adult reconstruction community. In native knee flexion, the PCL is comprised of an anterolateral and posteromedial bundle that work together to limit posterior tibial translation and allow adequate femoral rollback for deep flexion. In the arthritic knee, the PCL can often become dysfunctional and attenuated, which led to the development of posterior stabilized (PS) TKA bearing options. PS TKAs implement a cam-post construct to functionally replace a resected PCL. While PS designs may facilitate balancing knees with significant deformity, they are associated with complications such as postfracture, increased wear, and patellar clunk/crepitus. In recent years, newer designs have been popularized with greater degrees of congruency and incorporation of medial and lateral pivoting to better recreate native knee kinematics. The American Joint Registry has confirmed the recent predilection for ultra-congruent and cruciate-retaining TKA inserts over PS TKAs during the last decade. Studies have failed to identify an overall clinical superiority between the cruciate substituting and sacrificing designs. The literature has also failed to identify clinical consequences from PCL resection with modern, more conforming TKA designs. In this article, we review modern PCL sacrificing designs and discuss the impact of each on the kinematics after TKA. We also will delineate the role of the PCL in modern TKA in the hopes to better understand the recent surge in sacrificing but not substituting knee implants.

## Introduction and background

There have been significant changes in total knee arthroplasty (TKA) implant design over recent decades. Initial constructs focused on recreating the hinge joint of the knee, paying little attention to surrounding soft tissues [1]. In the 1970s, research elucidated the impact the anterior cruciate ligament (ACL) and posterior cruciate ligament (PCL) have on the kinematics and stability of a reconstructed knee [2]. While the ACL is commonly resected during modern TKA, there is debate on the role and utility of PCL retention in TKAs.

It is the surgeon’s decision whether to sacrifice or retain the PCL during primary TKA. From that choice, a second decision is made about whether to substitute the PCL (which determines the type of femoral component utilized). Cruciateretaining (CR) designs allow the PCL to remain intact, while posterior sacrificing designs manage the PCL with either a cam-post mechanism (posterior stabilized [PS]) or a more congruent insert to recreate its function, the latter maintaining similar characteristics to a CR femoral component. Theoretically, CR inserts preserve native knee kinematics with increased stability and proprioception from the retained PCL [3]. PS inserts were developed to overcome an often attenuated PCL in osteoarthritis to allow for improved range of motion (ROM), easier balancing, and more consistent femoral rollback in flexion; however, they’re also associated with potential complications such as increased fracture risk, cam-post wear, flexion instability, and patellar clunk [[4], [5], [6]]. Numerous investigations have sought to determine which design affords better kinematics, stability, and clinical outcomes [[7], [8], [9], [10]]. To date, the evidence has yet to overwhelmingly point to one design as being superior to the other.

More recently, ultra-congruent (UC) and other conforming (“pivot” or congruent designs) PCL sacrificing bearings have come to the market as alternatives to the traditional cam-and-post TKA. As resection of the PCL increases the flexion gap, design features such as a high anterior lip and deep trough for increased articular congruence, help guide femoral rollback and drive knee kinematics [[11], [12], [13]]. The aim of this article is to highlight the impact of each on the kinematics after TKA as well as delineate the role of the PCL in modern TKA in the hopes to better understand the recent surge in sacrificing, but not-substituting knee implants.

## Anatomy—function of PCL in TKA

**Figure 1:**
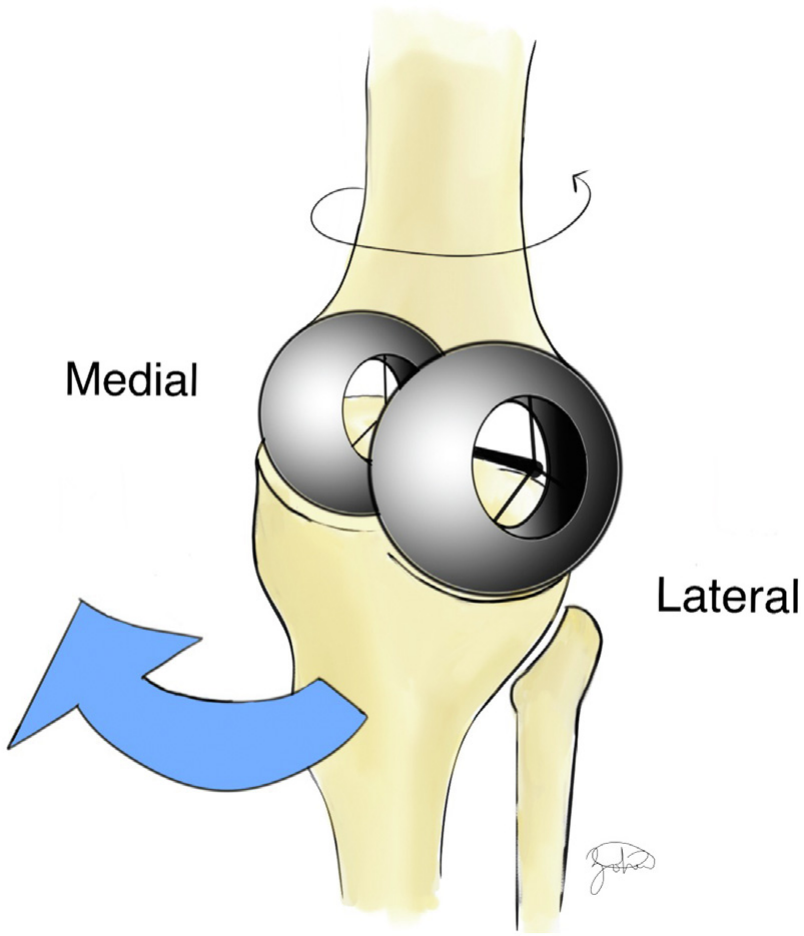
Illustration demonstrating the “big wheel-small wheel” construct. In this concept, the lateral femoral condyle (“big wheel”) has a large sagittal radius compared to the medial femoral condyle (“small wheel”), allowing the femur to travel father on the lateral tibial plateau compared to the medial side.

During native knee motion, the tibiofemoral articulation follows a predictable pattern. The smaller lateral femoral condyle translates posteriorly, while the larger, more static medial femoral condyle acts as a pivot, often described as a “screw-home mechanism” [2]. This dissimilarity in movement between the condyles fashions the distal femur in a “big wheel, little wheel” construct, allowing the tibia to internally rotate during knee flexion due to femoral external rotation (Fig. 1). A lack of femoral “roll-back”, would cause impingement at approximately 90° flexion. This posterior translation, primarily driven by the lateral condyle, allows for deep knee flexion.

**Figure 2:**
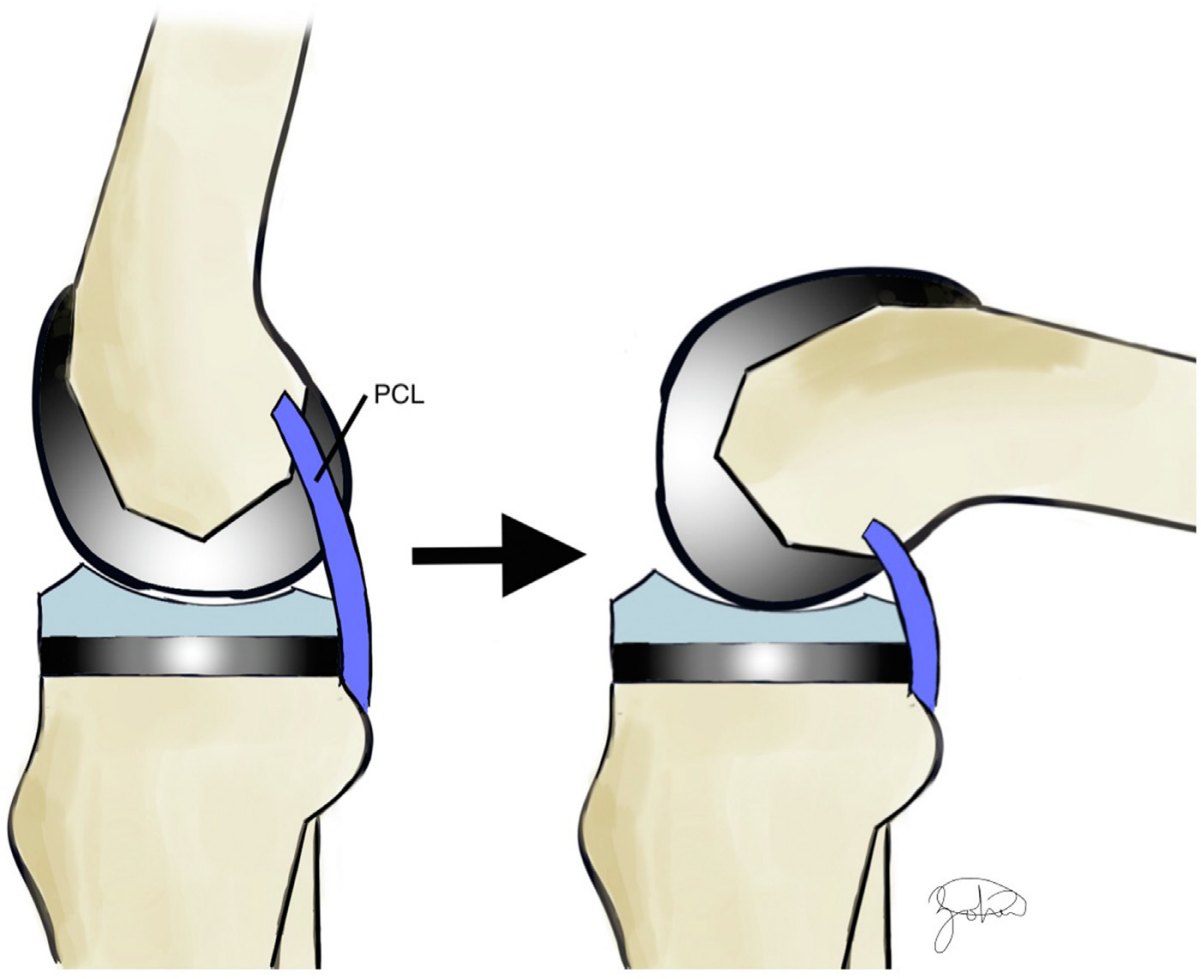
Illustration demonstrating PCL contribution to normal anatomic femoral rollback which tightens with knee flexion above 90° to promote femoral rollback and increase sagittal stability.

Natural biomechanics of the knee are altered during TKA. As modern TKA designs usually sacrifice the ACL, focus has turned to the PCL’s role to recreate native knee joint kinematics. The PCL is an intraarticular structure that originates from the anterolateral aspect of the medial femoral condyle and inserts 1 cm distal to the knee joint line along the posterior aspect of the tibial plateau [2]. It is comprised of an anterolateral and posteromedial bundle and functions as the primary restraint to posterior tibial translation during knee flexion [14]. In CR TKA, the PCL prevents posterior tibial subluxation during knee flexion, allowing for adequate femoral rollback [2,15] (Fig. 2). This controlled rollback is especially important in preserving knee ROM as the knee moves into deep flexion following TKA. Intraoperative assessment of the PCL is critical to ensure optimal TKA performance. Tests such as the “pull-out lift-off” (POLO) and “slide-back” have been developed to access PCL tension [16]. Furthermore, PCL attenuation and/or sacrifice will increase the flexion gap and should be accounted for when using PCL sacrificing but not substituting implants, along with other surgical technique considerations [13].

## Posterior cruciate ligament sacrificing designs

**Figure 3:**
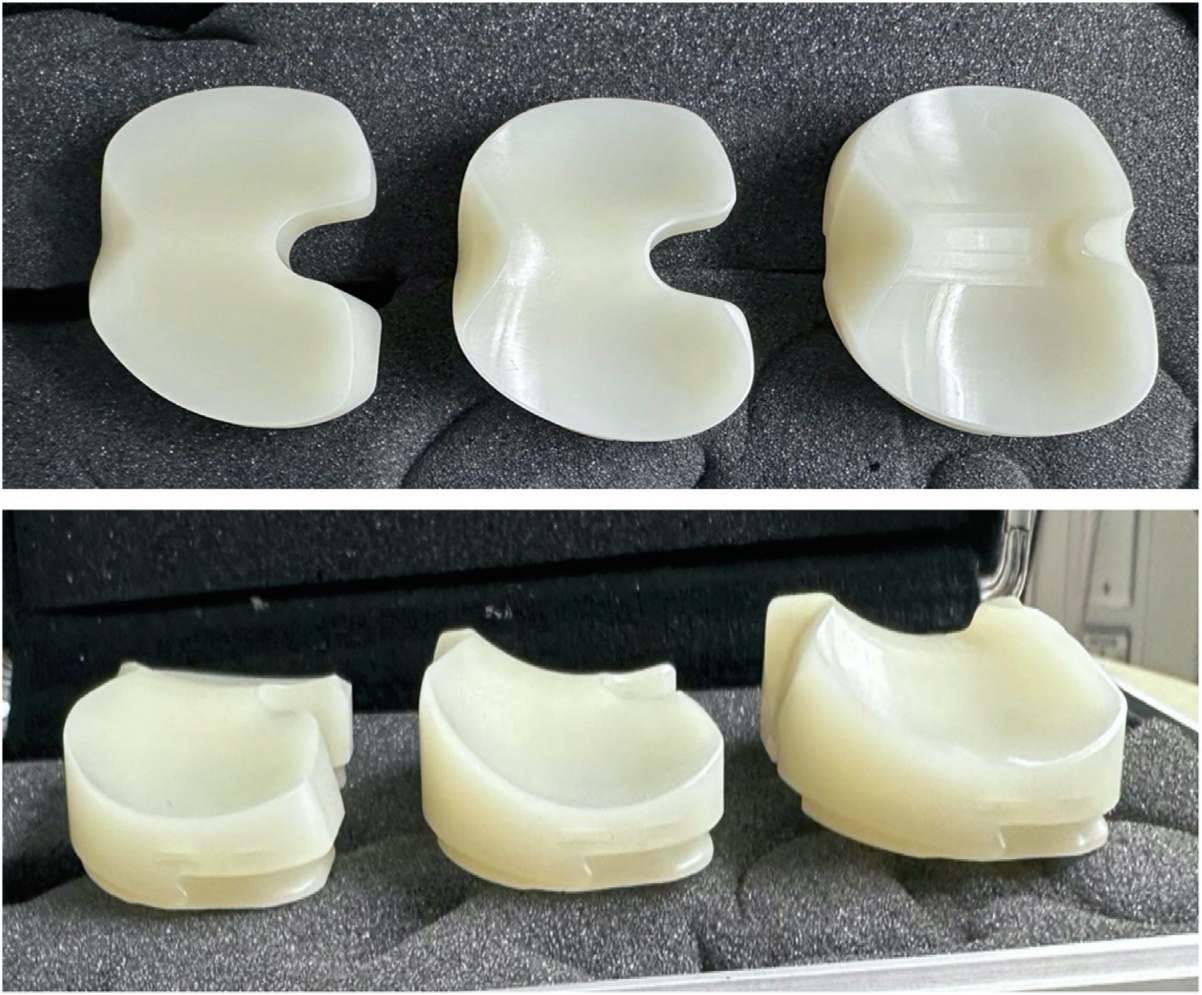
Standard cruciate retaining (CR) bearing compared to cruciate sacrificing designs. Left- CR standard implant, middle-medial congruent, right-ultra-congruent (UC) notice the closed posterior aspect of the UC polyethylene that requires PCL sacrifice.

Multiple PCL sacrificing TKA designs are available (Fig. 3). The general design characteristics, in addition to key implant advantages and disadvantages are summarized in Table 1. Most commonly used PCL sacrificing inserts are fixed-bearing, where the polyethylene insert is locked into the tibial tray. Other inserts, known as mobile-bearing or rotating-platform, allow for movement between the insert and tibial tray. These bearings can permit both anterior-posterior translation as well as internal-external rotation at the tray-insert interface, but some only facilitate rotation at this articulation [33]. Proposed advantages include reduced polyethylene wear and component loosening due to increased congruity and mobility in the tibiofemoral bearing surface [[42], [48]]. Mobile-bearing designs also offer the potential ability to self-adjust to accommodate surgical malalignment and improve patellofemoral mechanics [49,50]. Disadvantages include osteolysis, bearing dislocation, higher risk of patellar clunk (PS TKA only), and higher implant cost [[50], [51], [52], [53]]. Anterior lipped inserts have also been developed to provide support to a weak or partially released PCL by preventing posterior displacement of the tibia. While the lip effectively acts like a post, giving the bearing a “jump distance”, it may not be sufficient when the PCL is completely deficient or resected [38,54].

**Table 1:** Clinical summary of TKA bearing designs.

Design	Characteristics	Advantages	Disadvantages
Cruciate substituting/stabilizing (PS, MLC, BCS)	Femoral cam articulation against tibial polyethylene post PCL resected	Improved balancing with severe deformities and contractures [[17], [18], [19]] Increased and more reliable femoral rollback with deep flexion [20,21] Can be used in setting of compromised cruciate ligament [22]	Potential shearing at bone-cement interface [23] Femoral condylar fracture [24] Increased poly wear leading to fatigue failure (incidence .51%-1.2%) [[25], [26], [27], [28], [29]] Dislocation “Jumping the post” Patellar clunk (incidence 0%-7.5%- rare in modern PS design) [30,31] Possibly decreased survivorship due to long-term stress on tibia from the cam-post force transmission [32]
Ultra-congruent	Elevated anterior lip preventing posterior subluxation ± posterior lip Deep-dish conforming tibial troughs	Increased contact surface area with lower forces [11] Avoids patellar clunk	Restriction of femoral rotation leading to increased tibial stress [16,[33], [34], [35]] Decreased ROM [36,37] Tibial sagittal laxity leading to wear and osteolysis [2,5,[24], [38], [39], [40]] Anterior patellar translation leading to anterior knee pain and impaired extensor mechanism [41]
Medial pivot/congruent	Deep medial surface on tibial insert conforming to femoral condyle Pivot only - Single radius “ball and socket” Flat lateral compartment Anterior lip	Mimics native [42] knee kinematics Superior knee proprioception [43]	Potentially decreased ROM [44] Increased stiffness and revision rates [[27], [45]] Theoretically increased tibial baseplate force transmission due to ball-in-socket design
Lateral (dual) pivot	Spherical lateral tibial compartment Conforming medial tibial compartment with deeper flexion	Increased lateral conformity in extension and laxity in flexion to increase femoral rollback Improved stability in early phases of knee motion Improved patient satisfaction [46]	Abnormal instability mid-flexion [47] Limited medial femoral condylar movement [47] Limited internal rotation of tibia [47]

TKA, total knee arthroplasty; PS, posterior-stabilized; MCL, midlevel constraint; BCS, bicruciate stabilizing; PCL, posterior cruciate ligament; ROM, range of motion.

### Posterior substituting (cam-post)

**Figure 4:**
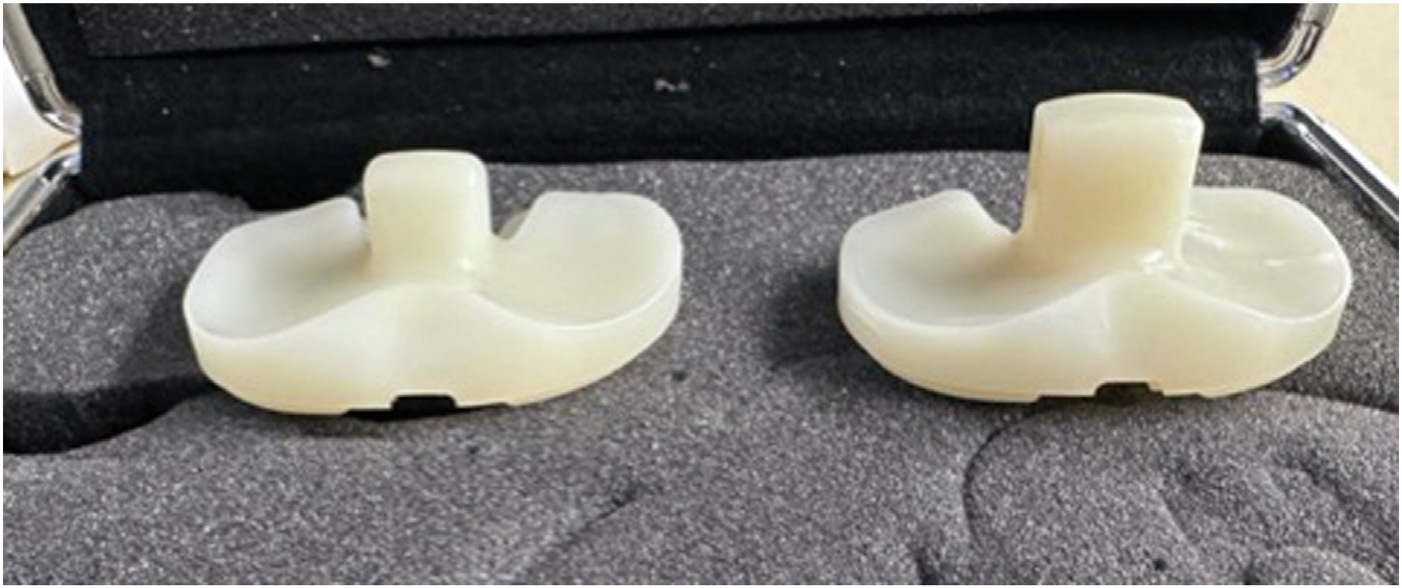
Left- Standard posterior stabilized (PS) insert; Right- midlevel constraint bearing providing increased varus-valgus stability.

**Figure 5:**
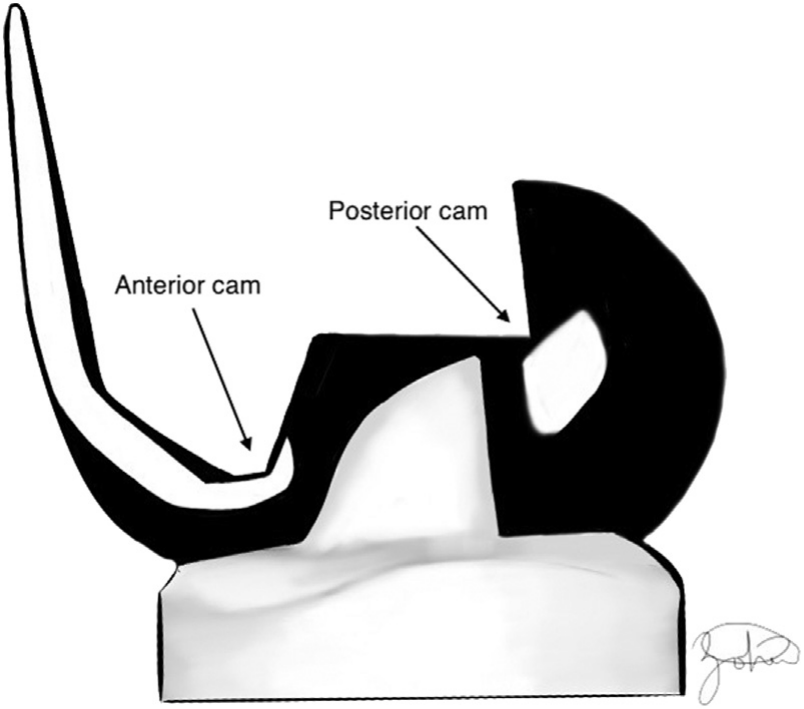
Illustration of bicruciate stabilizing implant with asymmetric post utilizing an anterior and posterior cam.

To prevent posterior subluxation of the tibia and improve knee ROM, the posterior stabilized (PS) knee was designed [15]. PS femoral components use a cam that articulates with a central post from the tibial polyethylene component that acts as a functional substitute for the PCL. This mechanism promotes femoral rollback on the tibia in flexion and also leads to compression at the bone-cement prosthesis interface; however, excessive force can also indicate an unbalanced knee [15]. Midlevel constraint (MLC) articular bearings utilize a similar but wider cam-post to provide increased varus and valgus and rotational stability (specific degrees of freedom vary for each company’s implants) [55] (Fig. 4). Some MLC bearing also utilize increased concavity at the medial and lateral plateau articulation; however, this is manufacturer specific. Study outcomes between PS and MLC TKA are summarized in Table 2. Lastly, bicruciate stabilizing (BCS) implants use an asymmetric cam-post mechanism, to substitute for both the ACL and PCL. In contrast to standard PS and MLC bearings, BCS implants have an asymmetric and medially conforming tibial plateau (Fig. 5).

**Table 2:** Summary of comparative studies between PS and MLC TKA.

Study	Number	Study type	Result	Level of evidence
King et al. (2014) [56]	68	Retrospective case-controlled study to determine effect of increased constraint on postoperative ROM	No difference in final ROM, pain relief, or function after TKA	III
Deshmukh et al. (2016) [57]	468	Retrospective cohort study to assess clinical outcomes between PS and MLC TKA	No difference in ROM, KSS score, radiographic outcomes, and revision rates	III
Moussa et al. (2017) [58]	1634	Retrospective cohort study to compare revision rates after PS and MLC TKA	Overall revisions rare in both groups but statistically significant higher revision rate in MLC cohort	III
Puah et al. (2018) [59]	76	Case-control study comparing clinical and functional outcomes between PS and MLC TKA	No difference in final knee ROM, Oxford Knee Score, and SF-36 scores.	III
Konopka et al. (2018) [60]	72	Implant retrieval analysis comparing wear patterns between PS and MLC TKA	Increased damage scores for retrieved MLC posts as well as significantly greater surface deviation in the posterior and medial post regions	III
Dubin et al. (2020) [61]	153	Retrospective review comparing PROMs, alignment correction, and revision rate between MLC and PS TKA	No difference in final ROM, anterior knee pain, pain VAS, or revision rates. MLC group had significantly greater improvement in PROMs	III
Colyn et al. (2022) [62]	60	Retrospective cohort study of patients with preoperative varus alignment >8 comparing clinical and functional outcomes between MLC, PS, and CR TKA.	Significantly better ML instability scores, KSS, and UCLA-activity score in MLC group compared to both the PS and CR cohort.	III

PS, posterior stabilized; MLC, mid-level constraint; TKA, total knee arthroplasty; ROM, range of motion; KSS, Knee Society Score; SF-36, short form (36) health survey; PROM, patient-reported outcome measure; VAS, visual analog scale; CR, cruciate retaining; UCLA, University of California Los Angeles Activity Scale.

### Ultra-congruent design

UC TKA is characterized by inserts that have an elevated anterior lip and a deep-dish conforming surface of the tibial component [11,14]. Using a more congruent articulation increases the articulating surface contact area and circumference to allow for greater knee stability during flexion and stair climbing [63,64]. This increased contact surface area allows for decreased contact forces and allows femoral rollback throughout the knee ROM [11]. The thick anterior lip on the tibial insert essentially functions as a PCL by preventing the tibia from posterior subluxation during knee flexion as well as a raised posterior lip to replicate the function of the ACL. While eliminating the cam-post theoretically causes less stress transfer to the tibial baseplate; the elevated posterior lip may decrease the maximal ROM; however, this is likely not clinically significant [65].

### Pivot designs

Greater insight into physiologic knee kinematics led to the advent of pivot models, which aimed to decrease the amount of shear forces on the tibial surface as well as increase the rotational freedom of the femur. While a true pivot implant has a single constant femoral radius both medially and laterally, alternate medial congruent implants have also been developed to replicate the natural “medial pivoting” of the knee but cannot be considered a true pivot-design implant and are labeled medial congruent implants. Clinical outcome studies for pivot designs are summarized in Table 3.

**Table 3:** Summary of clinical outcomes for pivot TKA.

Study	Number	Study type	Result	Level of evidence
Macheras et al. (2017) [66]	325	Retrospective review to assess long-term clinical and radiographic outcomes after MP TKA	Significant improvements in KSS, WOMAC, SF-12, and Oxford Knee Score. Survival analysis showed success rate of 98.8% at 17 years.	III
Vecchini et al. (2012) [67]	172	Retrospective review to evaluate clinical and radiographic results of MP TKA at mean follow-up of 7 years	Significant improvements in KSS, ROM, and pain. Survivorship analyzed showed success rate of 98.6%	IV
Youm et al. (2014) [68]	120	Retrospective cases series analyzing clinical and radiographic results in MP TKA with minimum 5-year follow-up	Increased ROM, improved post-operative tibiofemoral angles, and KSS and WOMAC scores. Seven-year survival rate was 98.1%.	IV
Schmidt et al. (2014) [69]	421	Retrospective case series to evaluate midterm clinical and radiographic outcomes after MP TKA	Significant improvements in PROMs and ROM. Component survivorship was 96.6% at 5 years.	IV
Ueyama et al. (2020) [70]	257	Retrospective case series investigating clinical results in MP TKA with minimum 10-year follow-up.	Significant improvements in both PROMs and ROM. Survival rate for reoperation and revision at 10 years was 96.3% and 98.4% respectively	IV
Cacciola et al. (2020) [71]	315	Retrospective review to evaluate survivorship and clinical outcomes of MP TKA with minimum 5-year follow-up.	Statistically significant improvements in all objective and subjective outcomes measurements. Survivorship at 5 years was 98.2%	IV
Indelli et al. (2021) [72]	79	Retrospective review to evaluate the clinical outcome of MC TKA in valgus knee with 2-year minimum follow up.	Statistically significant improvements in final ROM as well as knee valgus ankle. 99% had good or excellent KSS score.	IV
Meding et al. (2022) [73]	232	Retrospective case series to determine early clinical and radiographic results of a press-fit LP TKA	All but 2 patients reported either mild or no pain with activity. 3 knees (1.3%) were revised.	IV

TKA, total knee arthroplasty; MP, medial pivot; MC, medial congruent; KSS, Knee Society Score; ROM, range of motion; WOMAC, Western Ontario and McMaster Universities Osteoarthritis Index; PROM, patient-reported outcome measure; SF-12, short form (12) health survey; LP, lateral pivot.

#### Medial pivot

**Figure 6:**
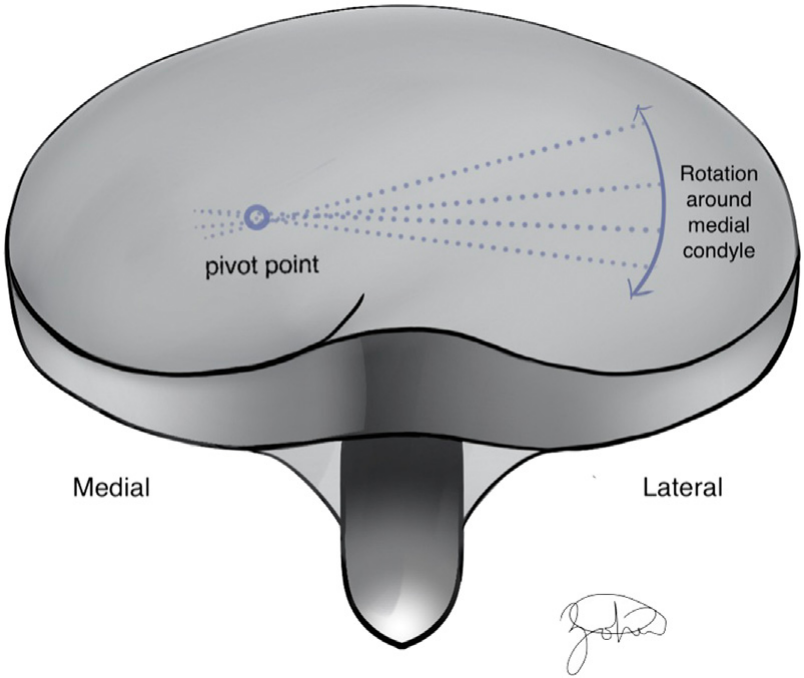
Illustration depicting a medial pivot design. The ball-in socket conformity allows for a medial pivot, permitting AP translation of the lateral condyle during flexion and extension.

**Figure 7:**
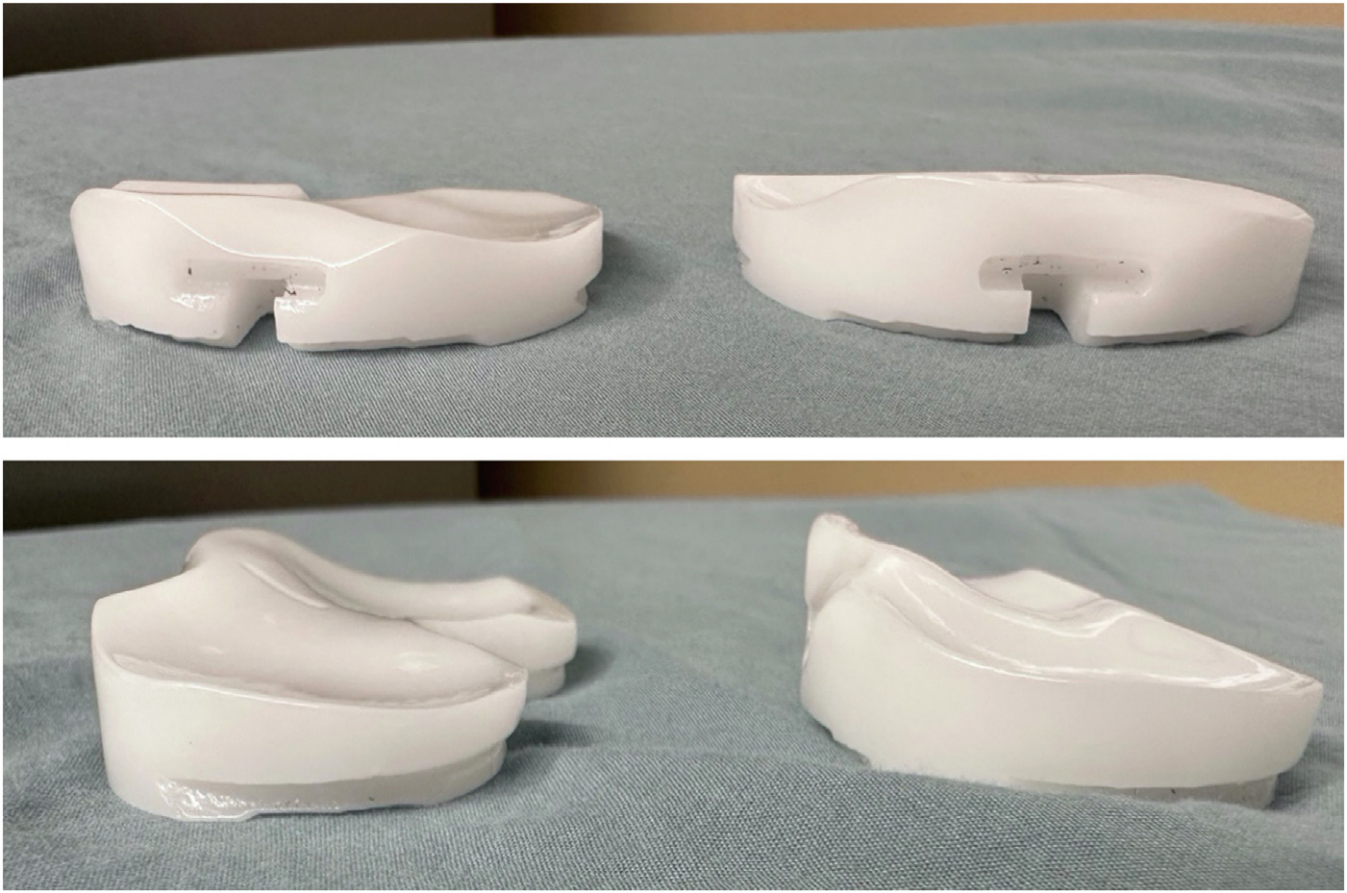
Images of a medial pivot design polyethylene. Left-cruciate-retaining medial congruent implant, and right-“true” medial pivot implant. The medial side is essentially a ball and socket with an elongated posterior medial lip to lock the medial side of the knee in place, while the lateral side is flatter affording more rollback in this medial pivot design.

During native knee flexion, the lateral femoral condyle slides posteriorly on the tibial plateau while a pivoting movement is observed in the medial compartment between the medial femoral condyle and the tibial plateau. The anatomy of the tibial plateau (medial plateau is concave and the lateral plateau convex) allows for this observed motion and creates a knee that is inherently more stable on the medial side. Medial pivot (MP) TKA designs try to mimic this physiological “medial pivoting” by having greater medial conformity provided by a concave surface on the medial compartment of the tibial insert [[44], [74]]. Typically, the single radius of the design of the femur will then function like a “ball and socket” in the concave medial surface of the polyethylene. The insert has an anterior lip that stabilizes the knee and resists posterior subluxation of the tibia during knee ROM, functionally replacing the PCL. The lateral compartment of the insert is flatter and allows for the lateral condyle to move unrestricted along an arcuate path during knee flexion [74] (Fig. 6). Medial congruent designs have similar kinematics but lack a true pivot due to the size difference between the medial and lateral femoral condyle (Fig. 7).

#### Lateral (dual) pivot

Recent interpretation of native knee kinematics has found a more complex pattern of differing pivot motions with various flexion ranges during knee ROM [75,76]. While there continues to be support for a medial pivot pattern in deep flexion, it is now believed that early flexion in an ACL-intact native knee is characterized by a lateral-pivot pattern [76,77]. These findings led to the development of dual-pivot TKA, which has a lateral tibiofemoral compartment that is spherically conforming in extension that progressively becomes more lax in flexion to allow the femoral condyle to translate posteriorly [78]. The congruence in the early phase of knee motion allows for greater stability during the majority of daily activities, such as walking and climbing stairs [79]. Conversely, the decreased conformity during greater knee flexion allows for a medial pivot pattern that is necessary for activities that require deep flexion.

## Do you need to substitute for the PCL?

The current interest in PCL sacrificing implants can partly be attributed to the inability of PS implants to recreate natural knee kinematics, causing continued dissatisfaction among patients [80]. Several studies have since evaluated differences in clinical outcomes between PS and cruciate sacrificing implant designs (Table 4). In an early randomized prospective study, Laskin et al. evaluated 176 patients with either a cam-post PS TKA or a UC design TKA. The PCL was resected in all patients, and there was no statistical difference in any of the measured variables, which included ROM, ability to ascend or descend stairs, outcome scores, stability or anterior knee pain [11]. These findings were supported in another randomized control study by Lutzner et al., who separated 127 patients into a UC and PS TKA group with 1-year postoperative follow-up. They found that while there was less sagittal translation and greater posterior femoral rollback in the PS TKA, there was no difference in ROM or patient-reported outcomes at final follow-up [85]. This was also seen in a study from Sur et al., which evaluated 28 patients who underwent bilateral TKA with a cam-post PS insert in one knee and an UC insert with the PCL resected in the contralateral knee. The UC group had about 8 mm more posterior tibial translation measured on stress radiographs, but this was not associated with any difference in functional outcome [84].

**Table 4:** Summary of comparative studies between cruciate sacrificing and substituting TKA.

Study	Number	Study type	Result	Level of evidence
Laskin et al. (2000) [11]	176	Randomized prospective study between UC and PS TKA. PCL resected in all patients	No difference in final ROM, ability to ascend/descend stairs, anterior knee pain, postoperative radiographic alignment, SF-36 score	I
Kim et al. (2021) [81]	100	Prospective randomized controlled trial comparing joint perception between UC and PS TKA in patients undergoing same-day bilateral TKA	Less noise generation in UC group. No difference in side preference, satisfaction, or Forgotten Joint Score between 2 groups. More sagittal laxity and less femoral rollback in UC group.	I
Akti et al. (2021) [82]	66	Randomized control trial to compare isokinetic performance and clinical outcomes between PS and UC TKA. PCL sacrificed in all patients	No difference in isokinetic performance or clinical outcomes at 1 year follow-up.	I
Hossain et al. (2011) [83]	82	Randomized control trial to compare objective and subjective outcomes between medially conforming ball-and-socket design and PS TKA	Greater final ROM and better SF-36 and Total Knee Function Questionnaire scores in medial conforming group. No difference in American Knee Society, WOMAC, and Oxford Knee Scores.	I
Sur et al. (2015) [84]	56	Prospective randomized trial comparing UC and PS TKA in patients undergoing one-stage bilateral TKA with minimum 5-year follow-up	No difference in final knee alignment, ROM, pain, or functional outcome (walking, stair climbing). Inferior Knee Society Score and increased anteroposterior laxity in UC group.	I
Lutzner et al. (2017) [85]	127	Randomized controlled trail between UC and PS TKA with 1-year follow-up. PCL resected in all patients.	No difference in ROM, Knee Society Score and Oxford Knee Score at 1-year follow-up. Increased sagittal translation and reduced posterior femoral rollback in UC implant.	I
Scott (2018) [9]	111	Prospective randomized trial comparing UC and PS TKA with 5-year follow-up. PCL resected in all patients	No difference in clinical outcome scores, ROM, or radiographic alignment. Increased mechanical sensation in PS group.	I
Bae et al. (2018) [86]	1797	Meta-analysis of 13 studies comparing clinical outcomes, kinematics, and ROM between UC and PS TKA.	No difference in clinical outcomes between groups. UC inserts demonstrated less posterior roll back and final ROM and more sagittal plane tibial laxity than PS insert.	I
Indelli et al. (2019) [87]	100	Randomized trial comparing patient satisfaction as well as clinical and radiographic outcomes in PS and medially congruent (MC) TKA. PCL resected in all patients	No difference in Oxford Knee Score and Knee Society Score. Increased ROM in MC group.	I
Fritzsche et al. (2018) [4]	40	Compared intraoperative kinematics, stability, and range of motion between native knee and UC and PS TKA in the same patient using a navigation system.	Native kinematics changed with both inserts. More posterior roll back and less anteroposterior translation with deep flexion in PS group. Slightly increased ROM in PS group (118 vs 123)	II
Biyani et al. (2017) [88]	82	Retrospective review comparing UC and PS TKA. PCL sacrificed in all patients	No difference in Modern Knee Society Score, walking and stair pain, UCLA activity level, or satisfaction at 1 year follow-up.	III
Parsley et al. (2006) [89]	209	Retrospective study comparing postoperative range of motion and functional outcomes between UC and PS TKA	Final knee ROM slightly higher in PS group (119.9 vs 116.7). No difference in Knee Score, Function Score, and satisfaction level.	III
Yacovelli et al. (2020) [10]	5970	Retrospective review comparing UC and PS TKA. PCL sacrificed in all patients	No difference in revision rate. PS group had higher KOOS Jr (69.9 vs 72.9) at 2 year follow-up.	III
Esposito et al. (2020) [90]	60	Gait analysis between normal knee and MP and PS TKA.	“Stiff knee pattern” seen in both groups but more pronounced in MP group.	IV

TKA, total knee arthroplasty; UC, ultra-congruent; PS, posterior stabilized; PCL, posterior cruciate ligament; ROM, range of motion; SF-36, short form (36) health survey; WOMAC, Western Ontario and McMaster Universities Osteoarthritis Index; UCLA, University of California Los Angeles Activity Scale; KOOS JR: Knee Injury and Osteoarthritis Outcome Score, Joint Replacement.

In a prospective randomized control study with a 5-year follow-up, Scott illustrated equivalent functional and radiographic outcomes in 111 patients treated with a PS or UC TKA [8,9]. Examining large institutional registry data, Yacovelli et al. compared outcomes of patients who underwent TKA using either a PS or UC insert. They evaluated 5970 patients and found comparable functional outcomes and revision rates between the 2 groups [10]. Kim et al. investigated joint perception between UC and PS TKA, with both cruciate ligaments resected in all cases [81]. In 50 patients undergoing same-day bilateral TKA using PS implant in one knee and UC insert in the other, no significant difference was observed in noise generation, patient side preference, and satisfaction [81]. Other clinical studies have also illustrated similar results comparing UC with traditional cam-post inserts [12,[82], [88], [89]].

Conversely, Fritzsche et al. measured intraoperative kinematics, stability, and ROM in 40 patients [4]. To avoid individual differences between patients, both UC and PS implants were tested in the same patient during surgery, and intraoperative measurements were performed using a navigation system. Kinematic changes observed in the UC insert included less femoral rollback and anteroposterior stability, leading to significantly less knee flexion of 123.1° compared to 127.6° in the PS insert group [4]. This decreased knee ROM was also illustrated in a recent meta-analysis comparing UC and PS inserts [86]. Both studies, however, failed to show any considerable clinical consequences despite altered knee kinematics and stability.

Pivot designs with resection of the PCL have also been directly compared to PS TKA. Esposito et al. performed kinematics, kinetics, and electromyography lower limb analysis during gait on 60 subjects (20 MP TKA, 20 PS TKA, and 20 healthy control) [90]. Both surgical groups were found to have a “stiff knee pattern”, however the medial pivot cohort showed inferior knee flexion and prolonged muscular activity of the rectus femoris compared to PS group [90]. Conversely, Hossain et al. randomized 82 patients to either receive a MP design knee vs a PS implant [83]. At the 2-year final follow-up, the MP group had significantly increased knee ROM and superior Total Knee Function Questionnaire Scores [83]. In a more recent randomized control study, Indelli et al. compared 50 patients with a PS knee vs a MP polyethylene insert [87]. At a minimum of 2-year follow-up, both groups had similar *Oxford Knee and Knee Society Scores*; however, there was a significantly increased ROM in the medial pivot group, which was not found to be clinically pertinent (120° vs 123°, *P* = .0089) [87].

Current clinical practice trends reflect the positive results illustrated in the literature. Between 2012 and 2020, the proportion of all TKAs performed using a CR bearing grew from 42.2% to 46.2%, and UC liners saw an increase in use from 4.9% to 9.1%, while PS TKAs decreased from 52.6% to 44.5% [46]. As more information comes to light on the clinical outcomes of PCL sacrificing inserts, it will be interesting to see if this trend toward increased utilization continues. Further, the role of retaining the PCL in some of the pivot-design implants requires further investigation as we continue to improve intra-operative balancing techniques using robotics and other forms of advanced technology.

## Should you retain the PCL?

Due to the nuances between CR and PS TKAs (tibial slope, flexion balance, and patella management), the decision to keep or resect the PCL is of critical importance. CR TKAs were designed with a posterior cut-out on the tibia and lack of a cam on the femoral component to allow retention of the PCL. Unlike in cruciate substituting concepts, the CR TKA depends on an intact and functional PCL to counteract posterior drag forces from the hamstring muscles and allow for femoral rollback during flexion [2]. The proposed benefits of keeping the PCL include fewer patella-related complications, increased comfort and ability to climb stairs, improved quadriceps strength, improved knee proprioception, preservation of femoral bone stock, and retention of near-natural kinematics [5]. However, with severe varus and valgus deformities in the knee, the PCL may become contracted and difficult to balance if kept, leading to instability and a higher incidence of postoperative pain, radiolucency, and stiffness [91,92]. Further, the relatively flat polyethylene required to allow PCL guided rollback has become a topic of debate due to less conformity and more focused areas of stress on the polyethylene, as well as paradoxical roll-forward in cases where the PCL is not fully functional.

With the advent of more congruent components and improved understanding of knee kinematics during flexion and extension, modern inserts allow surgeons to excise the PCL without necessarily substituting with a cam-post mechanism. There is controversy over whether patients truly require the PCL to achieve high function and satisfaction after TKA, and if modern designs sacrificing but not substituting the PCL offer superior outcomes to cruciate retaining TKAs (Table 5).

**Table 5:** Summary of comparative studies between cruciate sacrificing and retaining TKA.

Study	Number	Study type	Result	Level of evidence
Watanabe et al. (2013) [93]	74	Prospective study evaluating in vivo knee kinematics using LP TKA insert with or without the PCL	PCL sacrificing group illustrated greater knee ROM as well as more anterior femoral condylar positions	I
Roh et al. (2013) [94]	90	Randomized trial to evaluate kinematics and clinical outcomes between UC mobile-bearing TKA with or without the PCL	Increased varus rotation and femoral anterior translation in PCL preserving group. No difference in ROM, functional scores, and radiographic result. 3 revisions in PCL-preserving group.	I
Harman et al. (2014) [95]	159	Retrospective review of patients with dual pivot insert TKA who had PCL sacrificed or retained	No difference in Knee Society knee and function score, or sagittal laxity. Slightly increased ROM in PCL resect group.	II
Song et al. (2017) [96]	76	Prospective study to evaluate differences in functional outcome and in vivo kinematics between UC and CR TKA.	No difference in final ROM, HSS Score, WOMAC score, and KSS scores. No difference in mediolateral or anteroposterior laxity.	II
Meneghini et al (2022) [97]	40	Prospective study to evaluate 3D knee kinematics and PROMs in a dual-pivot TKA with and without PCL release	PCL release group had increased femoral AP translation and internal tibial rotation as well as inferior PROMs.	II
Kim et al. (2017) [98]	364	Compare clinical and radiographic results between MP and CR TKA in same patient. Average follow-up was 11 years	MP group had inferior knee scores, knee ROM, and patient satisfaction. There was higher complication rate in MP group. No difference in survival rate between inserts.	III
Peters et al. (2014) [5]	468	Retrospective review comparing Knee Society scores, radiographic results, complications rates and revision rates between UC and CR TKA.	CR group had high revision rate. No difference in other measured outcomes.	III
Stronach et al. (2019) [99]	161	Retrospective study to evaluate the effect of PCL retention in the same UC TKA implant	No difference in final ROM	III
Øhrn et al. (2019) [100]	60,566	National registry database study to evaluate differences in survival between MP and CR TKA	Increased revision risk in MP TKA group	III
Bae et al. (2011) [101]	137	Retrospective review to analyze results after TKA using a medial pivot insert with PCL retained and sacrificed	No difference in Knee Society knee and function score. No difference in femorotibial angle or ROM improvement between groups.	III
Mikashima et al. (2010) [102]	20	Examined in vivo kinematics and clinical outcomes between LP and CR TKA. PCL retained in all patients.	LP TKA had greater knee ROM, posterior lateral condyle translation, and tibial internal rotation.	III

TKA, total knee arthroplasty; LP, lateral pivot; CR, cruciate retaining; PCL, posterior cruciate ligament; ROM, range of motion; UC, ultra-congruent; MP, medial pivot; HSS, hospital for special surgery; WOMAC, Western Ontario and McMaster University Osteoarthritis Index; KSS, Knee Society Score; PROM, patient-reported outcome measure; AP: anterior-posterior.

Roh et al. reported the outcomes of 90 patients randomly assigned to receive UC TKAs with and without the PCL, showing no significant benefits of the PCL when it came to ROM captured by an intraoperative navigation system or patient-reported outcomes and radiographic results [94]. In fact, 3 out of the 42 (6.97%) knees with an UC liner and a retained PCL were revised for either instability or subluxation secondary to either attenuation or an overly tight PCL [94]. A similar study by Stronach et al. also failed to demonstrate a significant difference in postoperative ROM or revisions for instability between 161 patients randomly assigned to UC TKA with a retained or resected PCL [99].

When comparing outcomes between 468 UC and CR TKAs, Peters et al. found significantly more revisions in the CR group (21 cases) compared to the UC group (7 cases) [5]. The most common causes for aseptic revision were due to instability and loosening for patients with CR TKAs. Patient reported-outcomes and kinematics were comparable between the 2 cohorts [5]. Song et al. prospectively enrolled patients treated with either CR or UC TKA [96]. At a minimum of 3 years follow-up, they compared functional and radiographic outcomes in equal group sizes of 38 patients. Postoperative laxity assessments and patient-reported outcomes were improved in both groups; however, they were unable to determine superiority between the UC and CR inserts [96].

The contribution of the PCL to patient outcomes in MP TKA has also been analyzed. Bae et al. compared the outcomes for knees receiving the ADVANCE medial-pivot knee system (MicroPort Orthopedics Inc., Arlington, TN) with either the PCL retained (n = 67) or resected (n = 70) [101]. They determined there was no significant difference between the 2 groups in terms of final postoperative ROM or patient-reported outcome scores. A recommendation was given to resect the PCL if there was difficulty in obtaining tension during soft tissue balancing and there was a need to increase the flexion gap [101]. A systemic review performed by Cacciola et al. demonstrated similar survivorship rates between MP TKA and CR implants [103]. From the 18 studies ultimately included in their analysis, Knee Society Scores improved from 40.1 to 89.2 on average and ROM increased from 104.8° to 115.6° after MP TKA [103].

Clinically, there have been several studies raising concern over the benefits of MP inserts over CR designs in TKA. Analysis of both the Australian and Norwegian joint replacement national registries was performed by Øhrn et al. [100]. They determined there was an increased revision risk (hazard ratio (HR) of 1.5 [95% CI 1.2-1.7]) in MP designs compared to CR designs, per the Australian registry. The most common reasons for reoperation were prosthetic joint infection, patellar erosion, and aseptic loosening. The Norwegian registry was likewise associated with an increased HR of 1.5; however, this analysis was not statistically significant (95% CI 0.9-2.4) [100]. Similarly, Kim et al. investigated the long-term results of MP vs CR TKA in 182 patients [98]. At a minimum of 11 years after surgery, reported outcome scores, final ROM, and patient satisfaction were higher with knees receiving the CR insert over MP TKA. While radiographic survival rates were similar between the 2 groups, they observed a 26% (47 of 182 cases) complication rate in MP designs compared to 6.5% (11 of 182 cases) in the CR group [98]. As medial pivot designs improve over time, future studies may demonstrate improved outcomes compared to CR TKAs.

The role of the PCL has also been investigated in dual pivot TKA implants. Harman et al. compared dual pivot TKAs in 116 knees with an intact PCL to 43 with PCLs resected [95]. They noticed an average ROM of 127° in the dual pivot knees without PCLs, compared to 122° with the PCL. Interestingly, this did not correlate with patient-reported outcomes, as Knee Society Function Scores were on average 94 for patients retaining the PCL, compared to 87 without the PCL [95]. A similar study performed on 56 patients using dynamic radiographic analysis by Watanabe et al. observed removing the PCL allowed for increased anterior placement of the femoral condyles and external rotation in dual pivot TKAs. Final postoperative ROM was not significantly different whether the PCL was retained or not [93]. Finally, Meneghini et al. reviewed 40 patients who underwent dual-pivot TKA, in which the PCL was fully released in 21 (52.5%) of total cases. If excessive tightness was noted during gap balancing, the PCL was released to prevent anterior tibial insert and tray lift off. The PCL-release group had increased femoral anteroposterior translation (9.8 vs 5.5 mm) as well as greater maximum internal tibial rotation (6.2° vs −3.0°). The PCL released group also had inferior Knee Injury and Osteoarthritis Outcome Score for Joint Replacement and had a less natural feeling knee [97].

In vivo kinematics has also been compared between dual pivot and cruciate retaining TKAs. Mikashima et al. investigated the tibiofemoral positioning and orientation in various knee postures (standing, kneeling, max kneeling, and squatting) and clinical outcomes between 10 CR and 10 dual pivot TKAs [102]. While no difference was seen in Knee Society or Functional Scores, the dual pivot cohort exhibited on average 10° greater flexion in the various positions compared to the CR group. It should be noted, however, that the PCL was retained in all patients in this study [102]. A matched control study by Sandberg et al. and in vivo kinematic analysis by Meneghini et al. both demonstrated improved patient-reported Knee Society Function Scores compared to traditional noncongruent TKAs; however, direct comparison to CR TKAs were not conducted [78,104]. Future investigations are required to determine the long-term durability of new designs in relation to polyethylene wear, clinical outcomes, and overall survivorship. Surgeons should approach this new technology with cautious enthusiasm until the long-term data fully supports the currently mentioned short-term results.

## Conclusions

Management of the PCL and choice of polyethylene insert in TKA continue to be an ongoing debate among orthopedic surgeons. More new congruent inserts do not necessarily dependent on the PCL for knee function. While substitution of the PCL with a cam-post mechanism in PS TKA helps mimic physiologic femoral roll back to increase knee flexion, it is also associated with complications such as post breakage, additional bone resection, and patellar clunk syndrome. Conforming inserts were developed to overcome these limitations related with the cam-post mechanism. Studies do not indicate that substitution or even retention of the PCL is needed for improved clinical outcomes with these designs. As survivorship, clinical, and patient report outcomes between these implants continue to be disputed, it is ultimately the surgeon’s choice. Implant expertise, comfortability, and meticulous surgical technique must be underscored to assure the best outcomes for patients.

## Conflicts of interest

The authors declare there are no conflicts of interest.

For full disclosure statements refer to https://doi.org/10.1016/j.artd.2023.101130.

## Appendix A Supplementary data

Conflict of Interest Statement for Movassaghi

Conflict of Interest Statement for Patel

Conflict of Interest Statement for Levine

Conflict of Interest Statement for Ghulam-Jelani
